# Length of hospital stay after uncomplicated esophagectomy. Hospital variation shows room for nationwide improvement

**DOI:** 10.1007/s00464-020-08103-4

**Published:** 2020-10-26

**Authors:** Daan M. Voeten, Leonie R. van der Werf, Johanna W. van Sandick, Richard van Hillegersberg, Mark I. van Berge Henegouwen, on behalf of the Dutch Upper Gastrointestinal Cancer Audit Group

**Affiliations:** 1 Department of Surgery, Amsterdam UMC, Location AMC, Cancer Centre Amsterdam, University of Amsterdam, Amsterdam, The Netherlands; 2 Scientific Bureau, Dutch Institute for Clinical Auditing, Leiden, The Netherlands; 3 Department of Surgical Oncology, Antoni Van Leeuwenhoek Hospital, Amsterdam, The Netherlands; 4 Department of Surgery, University Medical Centre Utrecht, Utrecht, The Netherlands; 5 Department of Surgery, Amsterdam UMC, Location AMC, Room G4-180, Meibergdreef 9, 1105 AZ Amsterdam, The Netherlands; 6 Department of Surgery, Amsterdam UMC, Location AMC, Room G6-25, Meibergdreef 9, 1105 AZ Amsterdam, The Netherlands

**Keywords:** Esophageal carcinoma, Length of hospital stay, Readmission, Hospital volume, Hospital variation

## Abstract

**Background:**

Within the scope of value-based health care, this study aimed to analyze Dutch hospital performance in terms of length of hospital stay after esophageal cancer surgery and its association with 30-day readmission rates. Since both parameters are influenced by the occurrence of complications, this study only included patients with an uneventful recovery after esophagectomy.

**Methods:**

All patients registered in the Dutch Upper Gastrointestinal Cancer Audit (DUCA) who underwent a potentially curative esophagectomy between 2015 and 2018 were considered for inclusion. Patients were excluded in case of an intraoperative/post-operative complication, readmission to the intensive care unit, or any re-intervention. Length of hospital stay was dichotomized around the national median into ‘*short admissions’* and ‘*long admissions’*. Hospital variation was evaluated using a case-mix-corrected funnel plot based on multivariable logistic regression analyses. Association of length of hospital stay with 30-day readmission rates was investigated using the χ2-statistic.

**Results:**

A total of 1007 patients was included. National median length of hospital stay was 9 days, ranging from 6.5 to 12.5 days among 17 hospitals. The percentage of ‘*short admissions’* per hospital ranged from 7.7 to 93.5%. After correction for case-mix variables, 3 hospitals had significantly higher ‘*short admission’* rates and 4 hospitals had significantly lower ‘*short admission’* rates. Overall, 6.2% [hospital variation (0.0–13.2%)] of patients were readmitted. Hospital 30-day readmission rates were not significantly different between patients with a short length of hospital stay and those with a long length of hospital stay (5.5% versus 7.6%; *p* = 0.19).

**Conclusions:**

Based on these nationwide audit data, median length of hospital stay after an uncomplicated esophagectomy was 9 days ranging from 6.5 to 12.5 days among Dutch hospitals. There was no association between length of hospital stay and readmission rates. Nationwide improvement might lead to a substantial reduction of hospital costs.

**Electronic supplementary material:**

The online version of this article (10.1007/s00464-020-08103-4) contains supplementary material, which is available to authorized users.

Potentially curative treatment for locally advanced esophageal carcinoma consists of (neo)adjuvant chemo(radio)therapy followed by surgical resection. Esophagectomy is associated with significant post-operative morbidity. Approximately 65% of Dutch patients undergoing esophageal cancer surgery have a post-operative complication and 29% experience severe complications [1]. Post-operative complications are related to an increased length of hospital stay [2, 3]. In literature, median length of hospital stay after esophageal resection ranges from 8 to 14 days [3–6]. Prolonged length of hospital stay is a negative outcome of esophageal cancer surgery, not only for the patient but also for hospital finances [7–10]. In addition, complications have been related to higher readmission rates [11–15]. Hospital readmission also imposes a burden on patients and leads to an increase in hospital costs [7–9]. The relation between length of hospital stay and readmission has not been investigated in large cohorts of esophagectomy patients.

The ‘Dutch Upper GI Cancer audit’ (DUCA) aims to improve quality of care for surgically treated patients with esophageal or gastric cancer by benchmarking hospital results, and thus identifying variation in treatment, outcomes and clinical care pathways [16]. Reduction of hospital variation may enhance outcomes of care at a population level [17]. Next to quality of care, there is an increasing interest in value-based health care in oncology worldwide [18]. Comparing length of hospital stay and readmission rates after esophagectomy provides important insight into the efficiency of different post-operative care pathways and clinical practices in the Netherlands. Within that scope, this study aimed to analyze Dutch hospital performance in terms of length of hospital stay after esophageal cancer surgery and its association with 30-day hospital readmission rates. This study hypothesizes that hospital variation exists without higher readmission rates in hospitals with a short hospital stay. Since both parameters are influenced by the occurrence of complications, this study only included patients with an uneventful recovery after esophagectomy.

## Materials and methods

### Study design

For this population-based cohort study, data were retrieved from the DUCA dataset. This mandatory audit registers all patients with esophageal or gastric cancer undergoing surgery with the intent of resection since 2011. The DUCA dataset was verified; data completeness was estimated at 99.2% and outcome measure accuracy ranged from 95.3 to 100%.[19] As patients and hospitals are registered anonymously, ethical approval or informed consent was not needed according to Dutch Law. The current study protocol was approved by the DUCA scientific committee.

### Patient selection

All esophageal carcinoma patients undergoing potentially curative surgery between 2015 and 2018 were considered for inclusion. Patients were excluded in case of an intraoperative and/or post-operative complication, readmission to the intensive care unit, or any re-intervention. In order to give an overview of the current situation in the Netherlands, patients undergoing surgery in hospitals where esophageal cancer surgery was stopped before 2018 were excluded, to prevent redundant exclusion causing selection bias the 2015–2018 timeframe was chosen. In addition, patients were excluded if length of hospital stay was unknown/invalid.

### Outcomes

Length of hospital stay was calculated by subtracting the day of surgery from the day of discharge. In case date of discharge was before, or more than 200 days after the date of surgery, the entry was considered invalid. In the DUCA, short-term surgical outcomes are registered. Readmission is registered until 30 days after discharge from the hospital.

### Statistical analyses

Median length of hospital was reported at national and hospital level. Given its skewed distribution, length of hospital stay was dichotomized around the national median into ‘*short admissions*’ and ‘*long admissions’*. The exact median was added to the *short admission* group. Baseline characteristics between both groups were compared using the *χ*^2^ or fisher’s exact test. Hospital variation was evaluated using a case-mix corrected funnel plot [20, 21]. The expected number of *short admissions* for each hospital based on their case-mix was estimated using multivariable logistic regression analyses. The patient and tumor characteristics presented in Online Supplements Table 1 were used in the case-mix model. The observed number of *short admissions* divided by the estimated/expected (O/E ratio) was presented on the *y*-axis of the funnel plot, the *x*-axis showed the expected number. An O/E ratio larger than 1.0 indicated that more *short admissions* occurred than would be expected based on the hospital’s case-mix, whereas a ratio smaller than 1.0 indicated that less events occurred. Patients of outperforming and underperforming hospitals were pooled and 30-day readmission rates were compared using the *χ*2-statistic.

Univariable and multivariable logistic regression assessed possible factors associated with a *long admission*. Next to the baseline characteristics in Online Supplements Table 1, the following factors were investigated: hospital volume (the annual total esophagectomy hospital volume was assigned to each patient and thereafter dichotomized into < 40 or > 40), neoadjuvant therapy (chemoradiotherapy, none, chemotherapy), operative procedure (minimally invasive transthoracic, minimally invasive transhiatal, minimally invasive other, hybrid surgery, open transthoracic, open transhiatal and other open surgery), and anastomotic site (cervical, intrathoracic). Factors with a *p*-value < 0.1 in univariable analyses were added to multivariable analysis. To assess the association between length of hospital stay and 30-day readmission, the analyses above were repeated with readmission as dependent variable. Next to the variables described above, discharge during the weekend and length of hospital stay were added to this model.

### Sensitivity analyses

Based on previously published literature this study assumed complications impact length of hospital stay. This sensitivity analyses investigates this assumption in the DUCA dataset in order to prevent redundant exclusion of patients if the assumption does not apply. For these analyses, patients both with and without complications were included. Median length of hospital stay was reported at national level for the total cohort, patients with complications and patients with severe complications (grade Clavien Dindo grade 3 or higher) [22].

All p-values were two-sided, a *p* < 0.05 was considered statistically significant. Multicollinearity was assessed in all multivariable analyses using the variance inflation factor (VIF), a VIF ≥ 2.5 was considered indicative of multicollinearity. Missing values were analyzed in separate groups if exceeding 5%. Data were analyzed using R-studio version 1.2.5019, The R Foundation for Statistical Computing [23].

## Results

**Fig. 1:**
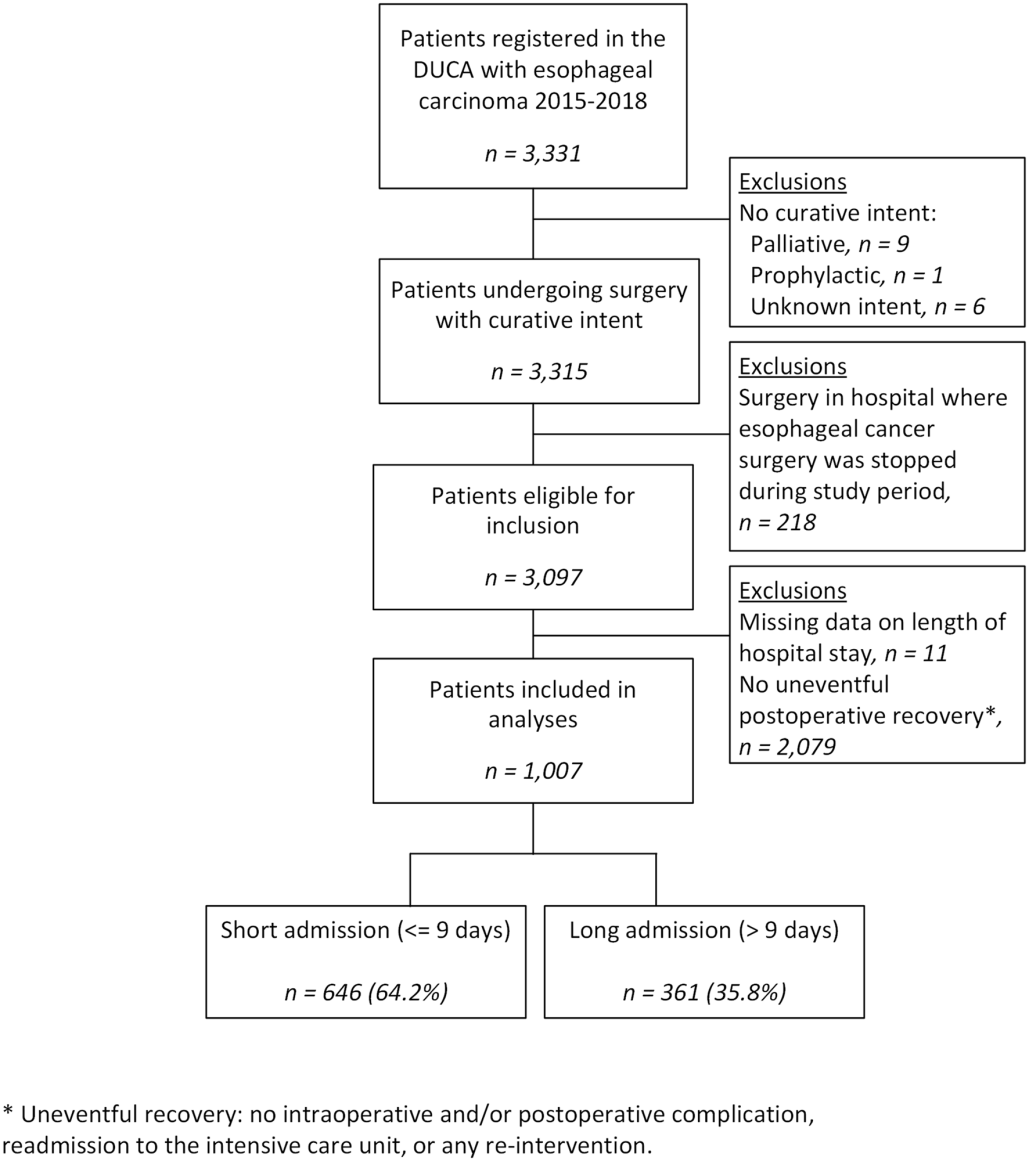
Flowchart of the study

**Fig. 2:**
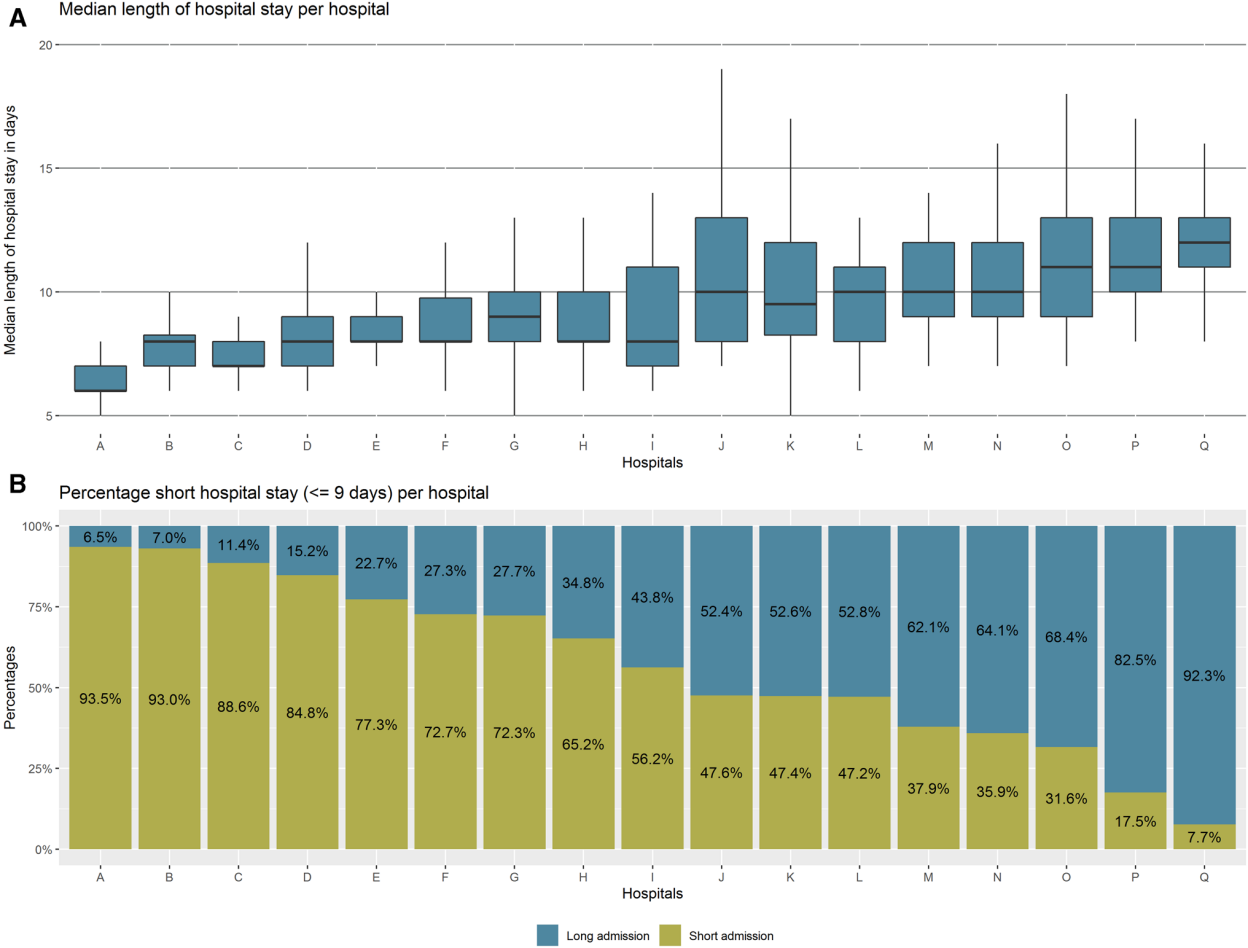
**A** Median length of hospital stay after uncomplicated esophagectomy per hospital. **B** Percentage of short (≤ 9 days) and long (> 9 days) hospital admissions after uncomplicated esophagectomy per hospital

**Fig. 3:**
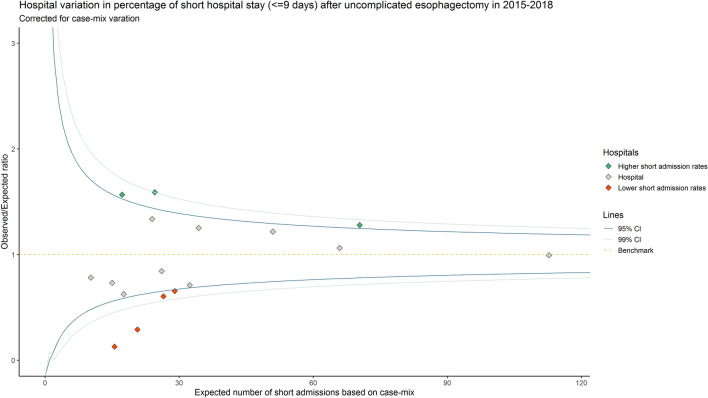
Case-mix-corrected funnel plot showing hospital variation in length of hospital stay after uncomplicated esophagectomy

A total of 1007 patients from 17 hospitals was included for analyses (Fig. 1). National median length of hospital stay after an uncomplicated esophagectomy was 9 days (IQR 7.0–11.0). Median length of hospital stay ranged from 6.5 (IQR 6.0–7.0) to 12.5 days (IQR 11.0–13.0) among the 17 hospitals (Fig. 2A). After dichotomization (≤ 9 days and > 9 days), 646 patients (64.2%) had a *short admission* and 361 patients (35.8%) had a *long admission*. Median length of stay was 8 days in the *short admission* group and 11 in the *long admission* group. The percentage of *short admissions* per hospital ranged from 7.7 to 93.5% (Fig. 2B). After correction for case-mix variables, the funnel plot showed 3 hospitals had significantly higher *short admission* rates and 4 hospitals had significantly lower *short admissions* rates than expected (Fig. 3).

### Clinical factors associated with prolonged length of hospital stay

Baseline patient, tumor, and treatment characteristics of patients with *short admissions* and *long admissions* are shown in Table 1.

**Table 1:** Baseline characteristics of patients with short (≤ 9 days) and long (> 9 days) admission after uncomplicated esophagectomy in 2015–2018

	Short admission (≤ 9 days)	Long admission (> 9 days)	Total	*P*-value (*χ*^2^/Fisher)
	*N* (%)	*N* (%)	*N* (%)	
Total	646 (64.2%)	361 (35.8%)	1007	
Sex				0.06
Male	524 (81.1%)	276 (76.5%)	800 (79.4%)	
Female	120 (18.6%)	85 (23.5%)	205 (20.4%)	
Missing	2 (0.3%)	0 (0%)	2 (0.2%)	
Age in years				0.53
< 65	291 (45.0%)	153 (42.4%)	444 (44.1%)	
65–75	304 (47.1%)	173 (47.9%)	477 (47.4%)	
> 75	51 (7.9%)	35 (9.7%)	86 (8.5%)	
Preoperative weight loss^a^				0.65
None	206 (31.9%)	128 (35.5%)	334 (33.2%0	
1–5	191 (29.6%)	97 (26.9%)	288 (28.6%)	
6–10	149 (23.1%)	79 (21.9%)	228 (22.6%)	
> 10	77 (11.9%)	44 (12.2%)	121 (12.0%)	
Missing	23 (3.6%)	13 (3.6%)	36 (3.6%)	
Body mass index (BMI)				0.32
< 20	37 (5.7%)	28 (7.8%)	65 (6.5%)	
20–25	328 (50.8%)	165 (45.7%)	493 (49.0%)	
26–30	208 (32.2%)	120 (33.2%)	328 (32.6%)	
> 30	73 (11.3%)	48 (13.3%)	121 (12.0%)	
ASA score^b^				0.73
I	132 (20.4%)	67 (18.6%)	199 (19.8%)	
II	401 (62.1%)	227 (62.9%)	628 (62.4%)	
III +	112 (17.3%)	67 (18.6%)	179 (17.8%)	
Missing	1 (0.2%)	0 (0%)	1 (0.1%)	
CCI^c^				0.18
0	363 (56.2%)	182 (50.4%)	545 (54.1%)	
1	141 (21.8%)	94 (26.0%)	235 (23.3%)	
2 +	142 (22.0%)	85 (23.5%)	227(22.5%)	
Previous esophageal or gastric surgery				0.06
No	635 (98.3%)	348 (96.4%)	983 (97.6%)	
Yes	9 (1.4%)	12 (3.3%)	21 (2.1%)	
Missing	2 (0.3%)	1 (0.3%)	3 (0.3%)	
Tumor location				< 0.01
Intrathoracic esophagus	481 (74.5%)	298 (82.5%)	779 (77.4%)	
Gastro-esophageal junction	165 (25.5%)	63 (17.5%)	228 (22.6%)	
Histology				0.20
Adenocarcinoma	526 (81.4%)	281 (77.8%)	807 (80.1%)	
Squamous cell carcinoma	93 (14.4%)	68 (18.8%)	161 (16.0%)	
Other	13 (2.0%)	6 (1.7%)	19 (1.9%)	
Unknown/missing	14 (2.2%)	6 (1.7%)	20 (2.0%)	
Clinical tumor stage^d^				0.19
T0-2	146 (22.6%)	98 (27.1%)	244 (24.2%)	
T3-4	470 (72.8%)	257 (71.2%)	727 (72.2%)	
Unknown/missing	30 (4.6%)	6 (1.7%)	36 (3.6%)	
Clinical node stage^d^				0.68
N0	255 (39.5%)	140 (38.8%)	395 (39.2%)	
N +	368 (57.0%)	215 (59.6%)	583 (57.9%)	
Unknown/missing	23 (3.6%)	6 (1.7%)	29 (2.9%)	
Neoadjuvant therapy				< 0.01
Chemoradiotherapy	566 (87.6%)	302 (83.7%)	868 (86.2%)	
None	23 (3.6%)	41 (11.4%)	64 (6.4%)	
Chemotherapy	57 (8.8%)	18 (5.0%)	75 (7.4%)	
Salvage surgery				1.00
No	618 (95.7%)	347 (96.1%)	965 (95.8%)	
Yes	8 (1.2%)	5 (1.4%)	13 (1.3%)	
Missing	20 (3.1%)	9 (2.5%)	29 (2.9%)	
Hospital volume (esophageal resections per year)				< 0.01
< 40	142 (22.0%)	134 (37.1%)	276 (27.4%)	
≥ 40	504 (78.0%)	227 (62.9%)	731 (72.6%)	
Surgical procedure				< 0.01
MI^e^ transthoracic	446 (69.0%)	236 (65.4%)	682 (67.7%)	
MI transhiatal	40 (6.2%)	19 (5.3%)	59 (5.9%)	
MI other	23 (3.6%)	4 (1.1%)	27 (2.7%)	
Hybrid	24 (3.7%)	34 (9.4%)	58 (5.8%)	
Open transthoracic	28 (4.3%)	49 (13.6%)	77 (7.6%)	
Open transhiatal	80 (12.4%)	17 (4.7%)	97 (9.6%)	
Open other	5 (0.8%)	2 (0.6%)	7 (0.7%)	
Anastomotic site				0.50
Cervical	251 (38.9%)	153 (42.4%)	404 (40.1%)	
Intrathoracic	361 (55.9%)	201 (55.7%)	562 (55.8%)	
None/other/missing	34 (5.3%)	7 (1.9%)	41 (4.1%)	
Hospital complication rate				< 0.01
< national median	385 (59.6%)	147 (40.7%)	532 (52.8%)	
> national median	261 (40.4%)	214 (59.3%)	475 (47.2%)	

^a^In kilograms

^b^American Society of Anesthesiologists Score

^c^Charlson Comorbidity Index

^d^In conformity with the 7th edition of the TNM rules for classification

^e^Minimally invasive

In multivariable logistic regression analyses, no neoadjuvant therapy, a hospital volume of under 40 esophagectomies per year and higher than average hospital complication rates were statistically associated with a *long admission* (Table 2). Compared to minimally invasive transthoracic surgery both open and minimally invasive transhiatal surgery and other minimally invasive surgery were associated with *short admissions.* Open transthoracic and hybrid surgery were associated with *long admissions*.

**Table 2:** Univariable and multivariable logistic regression analyses to assess the association of patient, tumor, and hospital characteristics with length of hospital stay after uncomplicated esophagectomy in 2015–2018

Factor	*N*	OR	Univariable analyses		aOR	Multivariable analysis	
			CI (95%)	*P*-value		CI (95%)	*P*-value
Sex							
Male	800	1			1		
Female	205	1.34	0.98–1.84	0.06	1.37	0.95–1.97	0.09
Age in years							
< 65	444	1					
65–75	477	1.08	0.83–1.42	0.57			
> 75	86	1.31	0.81–2.09	0.27			
Preoperative weight loss^a^							
None	334	1					
1–5	288	0.82	0.59–1.14	0.23			
6–10	228	0.85	0.60–1.21	0.38			
> 10	121	0.92	0.59–1.41	0.70			
Body mass index (BMI)							
< 20	65	1					
20–25	493	0.66	0.39–1.13	0.13			
26–30	328	0.76	0.45–1.32	0.33			
> 30	121	0.87	0.47–1.61	0.65			
ASA score^b^							
I	199	1					
II	628	1.12	0.80–1.57	0.53			
III +	179	1.18	0.77–1.80	0.45			
CCI^c^							
0	545	1			1		
1	235	1.33	0.97–1.82	0.08	1.31	0.92–1.86	0.13
2 +	227	1.19	0.86–1.65	0.28	1.13	0.78–1.63	0.52
Previous esophageal or gastric surgery							
No	983	1			1		
Yes	21	2.43	1.02–6.01	0.05	1.96	0.76–5.17	0.17
Tumor location							
Intrathoracic esophagus	779	1			1		
Gastro-esophageal junction	228	0.62	0.44–0.85	< 0.01	0.81	0.54–1.20	0.29
Histology							
Adenocarcinoma	807	1			1		
SCC	161	1.37	0.97–1.93	0.07	0.90	0.60–1.34	0.60
Other	19	0.86	0.30–2.21	0.77	0.48	0.15–1.37	0.19
Clinical tumor stage^d^							
T0-2	244	1					
T3-4	727	0.81	0.61–1.10	0.18			
Clinical node stage^d^							
N0	395	1					
N +	583	1.06	0.82–1.39	0.65			
Neoadjuvant therapy							
Chemoradiotherapy	868	1			1		
None	64	3.34	1.99–5.75	< 0.01	5.11	2.80–9.68	< 0.01
Chemotherapy	75	0.59	0.33–1.00	0.06	0.90	0.45–1.73	0.75
Salvage surgery							
No	965	1					
Yes	13	1.11	0.33–3.36	0.85			
Hospital volume (esophageal resections per year)							
< 40	276	1			1		
≥ 40	731	0.48	0.36–0.63	< 0.01	0.51	0.37–0.70	< 0.01
Surgical procedure							
MI^e^ transthoracic	682	1					
MI transhiatal	59	0.90	0.50–1.56	0.71	0.51	0.27–0.95	0.04
MI other	27	0.33	0.10–0.87	0.04	0.25	0.05–0.85	0.04
Hybrid	58	2.68	1.56–4.67	< 0.01	2.34	1.33–4.16	< 0.01
Open transthoracic	77	3.31	2.04–5.46	< 0.01	3.56	2.09–6.15	< 0.01
Open transhiatal	97	0.40	0.23–0.68	0.01	0.34	0.17–0.61	< 0.01
Open other	7	0.76	0.11–3.54	0.74	0.76	0.09–4.51	0.77
Anastomotic site							
Cervical	404	1					
Intrathoracic	562	0.91	0.70–1.19	0.50			
Hospital complication rate							
< National median	532	1					
> National median	475	2.15	1.65–2.74	< 0.01	2.18	1.62–2.94	< 0.01

^a^In kilograms

^b^American Society of Anesthesiologists Score

^c^Charlson Comorbidity Index

^d^In conformity with the 7th edition of the TNM rules for classification

^e^Minimally invasive

### Readmission

Of the 1007 included patients, 12 had missing data on readmission status. Overall, 62 of 995 patients (6.2%) were readmitted. The 30-day readmission rate ranged from 0.0 to 13.2% among hospitals. 30-day mortality occurred in one patient without readmission. There was no 30-day mortality among the 62 readmitted patients. Charlson Comorbidity Index, neoadjuvant therapy and surgical procedure were associated with readmission (Table 3). In addition, the readmission rate after discharge during the weekend was 10.2% (13 of 128) and 5.7% (49 of 867) after weekday discharge (*p* = 0.049). Hospital 30-day readmission rates were not significantly different between patients with a *short admission* and those with a *long admission* (5.5% vs 7.6%, respectively, *p* = 0.19). Given the small number of degrees of freedom, and small group sizes multivariable logistic regression analyses was not possible.

**Table 3:** Patient, tumor, and treatment characteristics of patients with and without 30-day readmission after uncomplicated esophagectomy in 2015–2018

	No readmission	Readmission	Total	*P*-value (χ^2^/Fisher)
	*N* (%)	*N* (%)	*N* (%)	
Total	933	62	995	
Sex				0.41
Male	744 (79.7%)	46 (74.2%)	790 (79.4%)	
Female	188 (20.2%)	15 (24.2%)	203 (20.4%)	
Missing	1 (0.1%)	1 (1.6%)	2 (0.2%)	
Age in years				0.55
< 65	412 (44.2%)	28 (45.2%)	440 (44.2%)	
65–75	439 (47.1%)	31 (50.0%)	470 (47.2%)	
> 75	82 (8.8%)	3 (4.8%)	85 (8.5%)	
Preoperative weight loss^a^				0.80
None	313 (33.5%)	17 (27.4%)	330 (33.2%)	
1–5	268 (28.7%)	18 (29.0%)	286 (28.7%)	
6–10	213 (22.8%)	14 (22.6%)	227 (22.8%)	
> 10	109 (11.7%)	9 (14.5%)	118 (11.9%)	
Missing	30 (3.2%)	4 (6.5%)	34 (3.4%)	
Body mass index				0.87
< 20	61 (6.5%)	3 (4.8%)	64 (6.4%)	
20–25	459 (49.2%)	31 (50.0%)	490 (49.2)	
26–30	305 (32.7%)	19 (30.6%)	324 (32.6%)	
> 30	108 (11.6%)	9 (14.5%)	117 (11.8%)	
ASA score^b^				0.76
I	185 (19.8%)	11 (17.7%)	196 (19.7%)	
II	584 (62.6%)	38 (61.3%)	622 (62.5%)	
III +	163 (17.5%)	13 (21.0%)	176(17.7%)	
Missing	1 (0.1%)	0 (0%)	1 (0.1%)	
CCI^c^				< 0.01
0	512 (54.9%)	26 (41.9%)	538 (54.1%)	
1	223 (23.9%)	12 (19.4%)	235 (23.6%)	
2 +	198 (21.2%)	24 (38.7%)	222 (22.3)	
Pre previous esophageal or gastric surgery vious esophageal or gastric surgery				1.00
No	910 (97.5%)	61 (98.4%)	971 (97.6%)	
Yes	20 (2.1%)	1 (1.6%)	21 (2.1%)	
Missing	3 (0.3%)	0 (0%)	3 (0.3%)	
Tumor location				0.21
Intrathoracic esophagus	726 (77.8%)	44 (71.0%)	770 (77.4%)	
Gastro-esophageal junction	207 (22.2%)	18 (29.0%)	225 (22.6%)	
Histology				0.19
Adenocarcinoma	743 (79.6%)	55 (88.7%)	798 (80.2%)	
Squamous cell carcinoma	154 (16.5%)	5 (8.1%)	159 (16.0%)	
Other	18 (1.9%)	1 (1.6%)	19 (1.9%)	
Unknown/missing	18 (1.9%)	1 (1.6%)	19 (1.9%)	
Clinical tumor stage^d^				0.29
T0-2	224 (24.0%)	18 (29.0%)	242 (24.3%)	
T3-4	678 (72.7%)	40 (64.5%)	718 (72.2%)	
Unknown/missing	31 (3.3%)	4 (6.5%)	35 (3.5%)	
Clinical node stage^d^				0.93
N0	366 (39.2%)	25 (40.3%)	391 (39.3%)	
N +	539 (57.8%)	36 (58.1%)	575 (57.8%)	
Nx	28 (3.0%)	1 (1.6%)	29 (2.9%)	
Neoadjuvant therapy				< 0.01
Chemoradiotherapy	815 (87.4%)	42 (67.7%)	857 (86.1%)	
None	54 (5.8%)	10 (16.1%)	64 (6.4%)	
Chemotherapy	64 (6.9%)	10 (16.1%)	74 (7.4%)	
Salvage surgery				0.20
No	894 (95.8%)	59 (95.2%)	953 (95.8%)	
Yes	11 (1.2%)	2 (3.2%)	13 (1.3%)	
Missing	28 (3.0%)	1 (1.6%)	29 (2.9%)	
Hospital volume (esophageal resections per year)				0.74
< 40	253 (27.1%)	18 (29.0%)	271 (27.2%)	
≥ 40	680 (72.9%)	44 (71.0%)	724 (72.8%)	
Surgical procedure				0.05
MI^e^ transthoracic	635 (68.1%)	41 (66.1%)	676 (67.9%)	
MI transhiatal	58 (6.2%)	1 (1.6%)	59 (5.9%)	
MI other	22 (2.4%)	3 (4.8%)	25 (2.5%)	
Hybrid	52 (5.6%)	4 (6.5%)	56 (5.6%)	
Open transthoracic	75 (8.0%)	2 (3.2%)	77 (7.7%)	
Open transhiatal	86 (9.2%)	9 (14.5%)	95 (9.5%)	
Open other	5 (0.5%)	2 (3.2%)	7 (0.7%)	
Anastomotic site				0.55
Cervical	375 (40.2%)	26 (41.9%)	401 (40.3%)	
Intrathoracic	526 (56.4%)	31 (50.0%)	557 (56.0%)	
None/other/missing	32 (3.4%)	5 (8.1%)	37 (3.7%)	
Weekend discharge				0.05
No	818 (87.7%)	49 (79.0%)	867 (87.1%)	
Yes	115 (12.3%)	13 (21.0%)	128 (12.9%)	
Length of hospital stay				0.19
Short admission (≤ 9 days)	604 (64.7%)	35 (56.5%)	639 (64.2%)	
Long admission (> 9 days)	329 (35.3%)	27 (43.5%)	356 (35.8%)	

^a^In kilograms

^b^American Society of Anesthesiologists Score

^c^Charlson Comorbidity Index

^d^In conformity with the 7th edition of the TNM rules for classification

^e^Minimally invasive

A total of 179 patients underwent surgery in the 3 outperforming hospitals (with more *short admissions*), of whom 10 were readmitted (5.6%). In the 4 underperforming hospitals (with fewer *short admissions*) 6.3% of 143 patients was readmitted, which was similar to the outperforming hospitals (*p* = 0.79).

### Sensitivity analyses

In total, 3086 patients underwent potentially curative surgery for esophageal carcinoma. Median length of hospital stay in this cohort was 11 days (IQR 8.0–18.0). Median length of hospital stay in patients with post-operative complications was 15 days (IQR 10.0–25.0). After a severe complication (Clavien Dindo grade 3 or higher), median length of hospital stay was 23 days (IQR 15.0–39.0). Online Supplements Table 2 shows associated factors with a *long admission* in this cohort.

## Discussion

This study showed hospital variation in length of hospital stay after uncomplicated esophageal resection for cancer. Median length of hospital stay ranged from 6.5 to 12.5 days among Dutch hospitals. In the current cohort, readmission rates after a short hospital stay and after a long hospital stay were comparable. However, readmission rates were higher in patients who were discharged in the weekend.

A retrospective cohort study using the NSQIP dataset, including over 3500 patients, reported a median hospital stay of 11 days after esophagectomy [3]. This is comparable to the median length of hospital stay in the sensitivity analyses of the current study. They did not report length of hospital stay for uncomplicated patients, but they did find complications to negatively affect length of hospital stay. This is similar to the results of our sensitivity analyses which showed median length of hospital stay was 15 days for complicated patients and 23 days for severely complicated patients. Several other retrospective studies also concluded that complications lead to longer hospital admissions [4, 24, 25]. To our knowledge, no literature is available on hospital variation in length of stay after uncomplicated esophagectomy. In the current study, median length of hospital stay had a 6-day difference between hospitals. Since only patients without complications were included, this difference is likely being caused by differences in clinical care pathways or discharge logistics. The hospitals with longer hospital stay might have difficulties in finding appropriate in-home care, or available nursing home beds. The timing of critical components of post-esophagectomy care (e.g., extubation, early mobilization, intensive care unit discharge, etc.) influences length of hospital stay [26]. In addition, several studies, including a meta-analysis of 18 studies, showed that the availability of a fast track or enhanced recovery after surgery (ERAS) protocol is associated with a shorter length of hospital stay [27–30]. The DUCA does not register ERAS protocol availability, therefore this statement could not be verified in the current study. Future DUCA research will focus on identifying the true reasons for hospital variation in length of hospital stay, afterwards focused improvement trajectories will be initiated.

Dutch upper gastrointestinal surgeons have yearly meetings in which different practices, logistics and clinical care pathways are discussed. These meetings aim to reduce hospital variation in upper gastrointestinal practices and outcomes. Discussing discharge logistics and clinical care pathways, or nationwide implementation of fast track recovery programs, may reduce hospital variation in length of hospital stay and eventually provoke a nationwide reduction in length of hospital stay and therefore hospital costs [8, 25]. Similar expert sessions were organized in the United Kingdom on different anastomotic techniques with positive results [31].

The Dutch guideline recommends neoadjuvant chemoradiotherapy for locally advanced esophageal cancer; at over 85% the compliance rate is high [32, 33]. Patients not receiving neoadjuvant therapy are probably not fit enough to receive intensive systemic therapy, which explains the longer hospital stay in these patients. In the current study, transthoracic surgery was associated with prolonged length of hospital stay which is in line with the results of a large meta-analyses comparing transhiatal and transthoracic surgery [34]. The opening of the thorax and more extensive lymph node dissection of transthoracic surgery might require longer in-hospital recovery independent of complications [35]. This study also showed shorter length of hospital stay in high-volume hospitals. This result corresponds with that of a systematic review and meta-analysis published in 2018 [36]. This study investigated the relationship between hospital volume and length of hospital stay after esophageal carcinoma surgery. A total of 75,383 patients were included and the authors concluded length of hospital stay was inversely related to hospital volume. In the current study, only uncomplicated patients were included, therefore the difference in hospital stay between low and high-volume hospitals was not caused by lower complication rates in the latter. High-volume hospitals might have invested more in efficient discharge logistics, clinical care pathways or ERAS protocols. More experienced nurses and residents may also play a role in faster discharge. This study also showed prolonged stay in hospitals with high complication rates, even for uncomplicated patients. This might be because of habit or fear of complications but might also reflect overlap with the volume-outcome relationship: shorter hospital stay and lower complication rates in high-volume hospitals [37].

Several studies investigated hospital readmission after esophagectomy, with readmission occurring in 11.2% to 18.6% [13, 14, 38–40]. These rates are higher than the 6.2% found in the current study but many of these studies found post-operative complications to be the biggest risk factor for a readmission. None of these reported on the effect of weekend discharge on readmission rates. Several studies investigated the effect of weekend discharge on readmission after other types of surgery than esophagectomy. None found higher readmission rates after weekend discharge [41–45]. All of these studies included patients both with and without complications. Given the results of the current study, even though not corrected for confounders, clinicians should be prudent in discharging (uncomplicated) esophagectomy patients during the weekend. Possibly, less continuity, and a decreased accuracy of discharge instructions, medication prescription, or home care planning could be the cause of a higher readmission rate after a weekend discharge.

This study has some limitations. It is not required to register in the DUCA when patients are ready for discharge; only the actual date of discharge is entered. Therefore, the underlying reasons for the identified hospital variation remain unclear. The DUCA only registers short-term surgical outcomes (during primary admission or, in case of discharge, in the first 30 post-operative days), therefore the impact of length of hospital stay on delayed complications could not be investigated. In addition, hospital readmissions are registered up to 30 days after primary discharge, the effect of length of hospital stay on longer-term readmission could not be analyzed. The sample of readmitted patients was small, correction for possible confounders using multivariable logistic regression was not possible and uncorrected results were presented.

In conclusion, based on these nationwide audit data, length of hospital stay after an uncomplicated esophagectomy varied significantly between hospitals and ranged from 6.5 to 12.5 days among Dutch hospitals. This variation indicates nationwide improvement could be achieved. This might lead to a substantial reduction of hospital costs. A short hospital stay was not associated with readmission rates. However, readmission rates were higher in patients who were discharged in the weekend.

## Electronic supplementary material

Below is the link to the electronic supplementary material.

Supplementary file1 (DOCX 28 kb)
