# Supplementary material for: Length of hospital stay after uncomplicated esophagectomy. Hospital variation shows room for nationwide improvement
Source: Surg Endosc. 2020 Oct 26;35(11):6344–57. doi: 10.1007/s00464-020-08103-4 (PMC8523439; doi:10.1007/s00464-020-08103-4)
Supplement: Supplementary file 1 — Supplementary file1 (DOCX 28 kb) [file 464_2020_8103_MOESM1_ESM.docx]

***Online supplements.*** *Table 1. Variables used in the case-mix model in hospital variation analyses.*

| Case-mix model | Variables | Options |
| --- | --- | --- |
| I | Sex | Male (ref) / Female |
| ii | Age in years | <65 (ref) / 65-75 / >75 |
| iii | American Society of Anesthesiologists score | I (ref) / II / III+ |
| iv | Charlson Comorbidity Index | 0 (ref)/ 1 / 2+ |
| v | Preoperative weight loss in kilograms | None (ref) / 1-5 / 6-10 / >10 |
| vi | Body Mass Index | <20 (ref) / 20-25 / 26-30 / >30 |
| vii | Tumor location | Intrathoracic (ref) / gastro-esophageal junction |
| viii | Clinical T-stage | T0-2 (ref) / T3-4 / Tx |
| ix | Clinical N-stage | N0 (ref) / N+ / Nx |
| x | Salvage surgery | No (ref) / yes |
| xi | Histology | Adenocarcinoma (ref) / squamous cell carcinoma / other |
| xii | Previous esophageal, gastric or hiatal surgery | No (ref) / yes |
| xiii | Each hospital’s complication rate during the study period | Lower than average (ref) / higher than average |

***Online supplements.*** *Table 2. Univariable and multivariable logistic regression analyses to assess the association of patient and tumor characteristics, and complications with length of hospital stay in patients undergoing esophagectomy in 2015-2018.*

|  |  | Univariable analyses | | | | | Multivariable analysis | | | |
| --- | --- | --- | --- | --- | --- | --- | --- | --- | --- | --- |
| Factor |  |  | OR | CI (95%) | P-value | OR | | CI (95%) | P-value |  |
| Sex |  |  |  |  |  |  | |  |  |  |
|  | Male | 2383 | 1 |  |  | 1 | |  |  |  |
|  | Female | 698 | 1.34 | 1.13 – 1.58 | <0.01 | 1.33 | | 1.07 – 1.65 | 0.01 |  |
| Age in years |  |  |  |  |  |  | |  |  |  |
|  | < 65 | 1281 | 1 |  |  | 1 | |  |  |  |
|  | 65 - 75 | 1461 | 1.23 | 1.06 – 1.43 | <0.01 | 1.36 | | 1.12 – 1.64 | <0.01 |  |
|  | > 75 | 342 | 1.85 | 1.45 – 2.36 | <0.01 | 2.11 | | 1.56 – 2.87 | <0.01 |  |
| Preoperative weight loss^a^ |  |  |  |  |  |  | |  |  |  |
|  | None | 1011 | 1 |  |  |  | |  |  |  |
|  | 1-5 | 895 | 0.95 | 0.79 – 1.13 | 0.55 |  | |  |  |  |
|  | 6-10 | 671 | 0.88 | 0.73 – 1.08 | 0.22 |  | |  |  |  |
|  | >10 | 360 | 0.87 | 0.68 – 1.11 | 0.26 |  | |  |  |  |
| Body Mass Index |  |  |  |  |  |  | |  |  |  |
|  | < 20 | 193 | 1 |  |  |  | |  |  |  |
|  | 20-25 | 1456 | 1.16 | 0.86 – 1.57 | 0.33 |  | |  |  |  |
|  | 26-30 | 1078 | 1.19 | 0.87 – 1.62 | 0.27 |  | |  |  |  |
|  | > 30 | 351 | 1.18 | 0.83 – 1.69 | 0.35 |  | |  |  |  |
| ASA score^b^ |  |  |  |  |  |  | |  |  |  |
|  | I | 484 |  |  |  | 1 | |  |  |  |
|  | II | 1867 | 1.48 | 1.21 – 1.82 | <0.01 | 1.16 | | 0.90 – 1.51 | 0.25 |  |
|  | III+ | 732 | 2.06 | 1.63 – 2.61 | <0.01 | 1.33 | | 0.97 – 1.81 | 0.07 |  |
| CCI^c^ |  |  |  |  |  |  | |  |  |  |
|  | 0 | 1558 | 1 |  |  | 1 | |  |  |  |
|  | 1 | 710 | 1.36 | 1.14 – 1.62 | <0.01 | 1.28 | | 1.01 – 1.61 | 0.04 |  |
|  | 2 + | 818 | 1.38 | 1.16 – 1.63 | <0.01 | 1.11 | | 0.89 – 1.38 | 0.37 |  |
| Previous esophageal or gastric surgery |  |  |  |  |  |  | |  |  |  |
|  | No | 2999 | 1 |  |  |  | |  |  |  |
|  | Yes | 74 | 1.10 | 0.69 – 1.75 | 0.68 |  | |  |  |  |
| Tumor location |  |  |  |  |  |  | |  |  |  |
|  | Intrathoracic esophagus | 2431 | 1 |  |  | 1 | |  |  |  |
|  | Gastro-esophageal junction | 641 | 0.74 | 0.62 – 0.89 | <0.01 | 0.92 | | 0.72 – 1.17 | 0.49 |  |
| Histology |  |  |  |  |  |  | |  |  |  |
|  | Adenocarcinoma | 2384 | 1 |  |  | 1 | |  |  |  |
|  | SCC | 588 | 1.35 | 1.13 – 1.62 | <0.01 | 0.91 | | 0.72 – 1.17 | 0.47 |  |
|  | Other | 62 | 1.26 | 0.76 – 2.10 | 0.37 | 0.90 | | 0.47 – 1.74 | 0.75 |  |
| Clinical Tumor stage^d^ |  |  |  |  |  |  | |  |  |  |
|  | T0-2 | 699 | 1 |  |  |  | |  |  |  |
|  | T3-4 | 2270 | 0.87 | 0.74 – 1.03 | 0.12 |  | |  |  |  |
| Clinical Node stage^d^ |  |  |  |  |  |  | |  |  |  |
|  | N0 | 1132 | 1 |  |  |  | |  |  |  |
|  | N+ | 1854 | 0.97 | 0.83 – 1.12 | 0.66 |  | |  |  |  |
| Neoadjuvant therapy |  |  |  |  |  |  | |  |  |  |
|  | Chemoradio-therapy | 2644 | 1 |  |  | 1 | |  |  |  |
|  | None | 225 | 2.00 | 1.51 – 2.66 | <0.01 | 2.40 | | 1.67 – 3.49 | <0.01 |  |
|  | Chemotherapy | 210 | 0.79 | 0.60 – 1.05 | 0.11 | 1.25 | | 0.83 – 1.86 | 0.28 |  |
| Salvage surgery |  |  |  |  |  |  | |  |  |  |
|  | No | 2905 | 1 |  |  |  | |  |  |  |
|  | Yes | 82 | 1.29 | 0.83 – 2.01 | 0.26 |  | |  |  |  |
| Hospital volume (esophageal resections per year) |  |  |  |  |  |  | |  |  |  |
|  | < 40 | 904 | 1 |  |  | 1 | |  |  |  |
|  | ≥ 40 | 2182 | 0.63 | 0.54 – 0.74 | <0.01 | 0.57 | | 0.46 – 0.70 | <0.01 |  |
| Surgical procedure |  |  |  |  |  |  | |  |  |  |
|  | MI^e^ transthoracic | 2027 | 1 |  |  |  | |  |  |  |
|  | MI transhiatal | 253 | 0.91 | 0.70 – 1.18 | 0.46 | 0.48 | | 0.34 – 0.68 | <0.01 |  |
|  | MI other | 89 | 0.31 | 0.18 – 0.50 | <0.01 | 0.39 | | 0.18 – 0.80 | 0.01 |  |
|  | Hybrid | 249 | 1.83 | 1.40 – 2.41 | <0.01 | 1.96 | | 1.41 – 2.74 | <0.01 |  |
|  | Open transthoracic | 214 | 1.91 | 1.43 – 2.58 | <0.01 | 2.54 | | 1.79 – 3.63 | <0.01 |  |
|  | Open transhiatal | 211 | 0.59 | 0.44 – 0.79 | <0.01 | 0.52 | | 0.34 – 0.76 | <0.01 |  |
|  | Open other | 42 | 0.53 | 0.27 – 0.99 | 0.05 | 0.72 | | 0.29 – 1.75 | 0.48 |  |
| Anastomotic site |  |  |  |  |  |  | |  |  |  |
|  | Cervical | 1354 | 1 |  |  | 1 | |  |  |  |
|  | Intrathoracic | 1541 | 0.83 | 0.72 – 0.97 | 0.02 | 0.82 | | 0.66 – 1.01 | 0.06 |  |
|  | None/other/missing | 191 | 0.51 | 0.37 – 0.69 | <0.01 | 0.59 | | 0.36 – 0.97 | 0.04 |  |
| Complications |  |  |  |  |  |  | |  |  |  |
|  | No | 1105 | 1 |  |  | 1 | |  |  |  |
|  | Yes | 1980 | 8.85 | 7.41 – 10.61 | <0.01 | 4.47 | | 3.64 – 5.52 | <0.01 |  |
| Severe complications^f^ |  |  |  |  |  |  | |  |  |  |
|  | No | 2156 | 1 |  |  | 1 | |  |  |  |
|  | Yes | 930 | 10.36 | 8.53 – 12.66 | <0.01 | 5.67 | | 4.54 – 7.13 | <0.01 |  |

**Legend:**

^a^ In kilograms

^b^ American Society of Anesthesiologists Score

^c^ Charlson Comorbidity Index

^d^ In conformity with the 7^th^ edition of the TNM rules for classification

^e^ Minimally invasive

^f^ Clavien Dindo grade III or higher
